# ﻿Pleomorphic *Dematiomelanommayunnanense* gen. et sp. nov. (Ascomycota, Melanommataceae) from grassland vegetation in Yunnan, China

**DOI:** 10.3897/mycokeys.98.107093

**Published:** 2023-07-25

**Authors:** Ying Gao, Tingfang Zhong, Jayarama D. Bhat, Antonio Roberto Gomes de Farias, Turki M. Dawoud, Kevin D. Hyde, Weiqiang Xiong, Yunju Li, Heng Gui, Xuefei Yang, Shixi Wu, Dhanushka N. Wanasinghe

**Affiliations:** 1 Center for Mountain Futures, Kunming Institute of Botany, Honghe 654400, Yunnan, China Center for Mountain Futures, Kunming Institute of Botany Kunming China; 2 School of Science, Mae Fah Luang University, Chiang Rai 57100, Thailand Mae Fah Luang University Chiang Rai Thailand; 3 Center of Excellence in Fungal Research, Mae Fah Luang University, Chiang Rai 57100, Thailand Key Laboratory of Economic Plants and Biotechnology and the Yunnan Key Laboratory for Wild Plant Resources, Kunming Institute of Botany, Chinese Academy of Sciences Kunming China; 4 Key Laboratory of Economic Plants and Biotechnology and the Yunnan Key Laboratory for Wild Plant Resources, Kunming Institute of Botany, Chinese Academy of Sciences, Kunming, 650201, China University of Chinese Academy of Sciences Beijing China; 5 University of Chinese Academy of Sciences, Beijing 100049, China King Saud University Riyadh Saudi Arabia; 6 Department of Botany and Microbiology, College of Science, King Saud University, P.O. Box 2455, Riyadh-11451, Saudi Arabia Vishnugupta Vishwavidyapeetam Gokarna India; 7 Biology Division, Vishnugupta Vishwavidyapeetam, Ashoke, Gokarna 581326, India Science and Technology on Aerospace Chemical Power Laboratory, Hubei Institute of Aerospace Chemotechnology Hubei China; 8 Science and Technology on Aerospace Chemical Power Laboratory, Hubei Institute of Aerospace Chemotechnology, Xiangyang, 441003, Hubei, China The State Phosphorus Resource Development and Utilization Engineering Technology Research Centre Kunming China; 9 The State Phosphorus Resource Development and Utilization Engineering Technology Research Centre, Yunnan Phosphate Chemical Group Co. Ltd, Kunming, China YTH Modern Agriculture Development Co. Ltd Kunming China; 10 YTH Modern Agriculture Development Co. Ltd, Kunming, China Center for Mountain Futures, Kunming Institute of Botany Yunnan China

**Keywords:** Asexual morph, Greater Mekong Subregion, molecular phylogeny, muriform, Pleosporales, sexual morph, taxonomy

## Abstract

During a survey of microfungi associated with grasslands and related vegetation types from Yunnan Province in China, various ascomycetous and coelomycetous fungi were isolated. This study reports the discovery of four strains of ascomycetous and coelomycetous fungi from dead stalks of *Hypericummonogynum* L. (Hypericaceae) and *Rubusparvifolius* L. (Rosaceae) in the Zhaotong region of Yunnan Province, China. The isolates were characterized using multi-locus phylogenetic analyses and were found to represent a new monophyletic lineage in Melanommataceae (Pleosporales, Dothideomycetes). This new clade was named as *Dematiomelanommayunnanense* gen. et sp. nov. which consists of both sexual and asexual morphs. The sexual morph is characterized by globose to subglobose ascomata with a central ostiole, cylindrical asci with a pedicel and ocular chamber, and muriform, ellipsoidal to fusiform ascospores. The asexual morph has synanamorphs including both brown, muriform macroconidia and hyaline, round to oblong or ellipsoidal microconidia. These findings contribute to the understanding of fungal diversity in grasslands and related vegetation types in Yunnan Province, China.

## ﻿Introduction

Melanommataceae is a species-rich family in the order Pleosporales and currently encompassing 351 species (Bánki et al. 2023) which have diverse lifestyles *viz.*, fungicolous, hyperparasitic, parasitic or saprobic (Tian et al. 2015; Hashimoto et al. 2017; Wijayawardene et al. 2017; Beenken et al. 2020; Hongsanan et al. 2020). The majority of species in this family have a wide distribution in temperate and subtropical regions and are commonly found on twigs or barks of various woody plants in terrestrial, marine, or freshwater habitats (Hyde et al. 2013; Tian et al. 2015). The latest treatment of the family by Wijayawardene et al. (2022a) accepted 35 genera in Melanommataceae. Except for *Asymmetricospora*, *Bicrouania*, *Calyptronectria*, *Exosporiella*, *Mamillisphaeria*, *Melanocamarosporium*, *Navicella* and *Nigrolentilocus*, all other genera have available sequence data for molecular comparisons.

Melanommataceae is a family of fungi that has been studied extensively, but few reports exist on its species found in China. Among the earliest reports are *Aposphaeriafugax* (Saccardo 1921; Wei and Huang 1939), *Aposphaeriapunicina* (Teng 1936), and *Melanommaglumarum* (Tai 1979). Subsequent studies have identified additional species, including *Camposporiumhyderabadense* (Matsushima 1980), *Byssosphaeriajamaicana* (Sivanesan and Hsieh 1989), *Melanommacucurbitarioideum* (Yuan and Barr 1994), and *Navicellaxinjiangensis* (Yuan and Barr 1994). More recent studies have introduced *Seifertiashangrilaensis* (Li et al. 2016), *Fusiconidiumaquaticum* (Li et al. 2017), *Alpinariarhododendri* (Thiyagaraja et al. 2020), and *Byssosphaeriaphoenicis* (Kularathnage et al. 2022). Despite these findings, there is still much to learn about the fungal diversity of Melanommataceae in China.

Grassland ecosystems are a vital component of the Earth’s land surface, covering an area of 52.5 million km2 and providing numerous ecosystem services (Bai and Cotrufo 2022). The plant species in this biome host various microorganisms, including fungi, with a broad spectrum of nutritional modes (Karunarathna et al. 2022). Grassland ecosystems support a high diversity of fungi and are likely to harbor numerous undescribed taxa (Hyde et al. 2020). However, human disturbance and climate change have been causing the rapid destruction and degradation of grasslands, leading to slow or non-existent recovery of biodiversity and essential functions (White et al. 2000; Chen et al. 2018; Bardgett et al. 2021; Lugato et al. 2021; Buisson et al. 2022; Zhu et al. 2022). Fungi are sensitive to environmental changes and global warming, which may be triggering the extinction of many species that cannot adapt fast enough to the rate of ecological change (Wanasinghe et al. 2022). In order to mitigate species loss and understand their ecological significance, extensive fungal sampling across various grasslands in different geographic regions is urgently required. Therefore, we are continuously surveying the grassland-associated microfungi in Yunnan, China. As a result, several strains of unknown species were isolated from different plant hosts.

This paper describes a fungus associated with *Hypericummonogynum* and *Rubusparvifolius* in the Zhaotong region as a new species in a new genus (*Dematiomelanomma*) within Melanommataceae, with its phylogenetic position being confirmed based on multi-locus phylogenetic analyses of ITS, LSU, SSU, *tef1-α* and *rpb2*. Furthermore, we compared it with the known genera in the family. This study provides insight into the grassland fungi in China and emphasizes that Zhaotong grasslands may have many undiscovered fungal resources waiting to be described.

## ﻿Materials and methods

### ﻿Sample collection and isolation

Specimens were collected from the dead wood of *Hypericummonogynum* L. (Hypericaceae) and *Rubusparvifolius* L. (Rosaceae) in Zhaotong, Yunnan, China, during autumn. The local environment in Zhaotong features Poaceae as the most abundant tree species and a typical plateau vegetation with a three-dimensional monsoon climate at a maximum elevation of ~4000 m (Pei 2022). Samples were taken to the laboratory in plastic Ziplock bags for observation and examination. Fungal specimens were rehydrated with tap water and examined using an Olympus SZ-61 dissecting microscope. Single spore isolation of both ascospores and conidia was conducted, and germinated spores were processed by following the methods described in Senanayake et al. (2020). Pure cultures were incubated at 26 °C for two weeks. The living cultures were deposited in the Kunming Institute of Botany Culture Collection (KUNCC), and duplicates were maintained in the China General Microbiological Culture Collection Center (CGMCC). Dried herbarium specimens (at room temperature) were deposited in the herbarium of the Kunming Institute of Botany Academia Sinica (HKAS). The Index Fungorum and Faces of fungi (FoF) numbers were obtained for the new taxa (Jayasiri et al. 2015; Index Fungorum 2023). Data from the Greater Mekong Subregion are deposited to the GMS database (Chaiwan et al. 2021).

### ﻿Morphological observations

Ascomata and conidiomata were hand-sectioned using a sterilized razor blade. Internal structures such as asci, ascospores, hamathecium tissues, conidiophores, and conidia were mounted on a slide in a drop of tap water using a sterilized needle to observe the micromorphological characteristics. These features were examined under a Nikon ECLIPSE Ni-U complex microscope with differential interference contrast (DIC) and phase contrast (PC) illumination. Images of microscopic structures were captured using a Nikon DS-Ri2 camera. Photo plates and measurements were processed using Adobe Photoshop CS6 Extended version 13.0.1 (Adobe Systems, CA, USA). Wherever possible, at least 30 measurements were taken. For morphological structures, mean, minimum, maximum and standard deviation were calculated. Structural dimensions are reported as mean ± standard deviation.

### ﻿DNA extraction, PCR amplification and DNA sequencing

Fungal mycelia grown on PDA for 2–3 weeks were scraped using a sterilized scalpel and transferred to 1.5 mL centrifuge tubes. The extraction of genomic DNA was performed using these fresh mycelia following the methods of Wanasinghe et al. (2016), using the Biospin Fungus Genomic DNA Extraction Kit (BioFlux, Hangzhou, P.R. China) following manufacturer guidelines. Also, genomic DNA from the fresh fruiting bodies was extracted using an E.Z.N.A. Forensic DNA Kit-D3591 (Omega Biotek, Inc) following the manufacturer’s protocol for further confirmation of our single spore isolations. The reference DNA for the polymerase chain reaction (PCR) were stored at 4 °C for regular use and at -20 °C for long-term usage.

The genomic DNA was used to amplify gene regions 18S small subunit rDNA (SSU), 28S large subunit rDNA (LSU), internal transcribed spacers (ITS), translation elongation factor 1-alpha (*tef*1-α) and RNA polymerase second largest subunit (*rpb*2) as described in Wanasinghe and Mortimer (2022). The total volume of PCR mixtures for amplification was 25 μL containing 8.5 μL ddH_2_O, 12.5 μL 2×F8FastLong PCR MasterMix (Beijing Aidlab Biotechnologies Co.Ltd), 2 μL of DNA template, 1 μL of each forward and reverse primers (stock of 10 pM). The PCR thermal cycle profiles for ITS, LSU, SSU and *tef1-α*: the thermal conditions included initial denaturation at 94 °C for 3 min, followed by 35 cycles of denaturation at 94 °C for 10 s; annealing temperatures at 55 °C for 15 s, elongation at 72 °C for 20 s, and final extension at 72 °C for 10 min. The PCR amplification condition of *rpb2* was set as denaturation at 95 °C for 3 min, followed by 35 cycles of denaturation at 95 °C for 45 s, annealing temperatures at 57 °C for 50 s, elongation at 72 °C for 90 s, and final extension at 72 °C for 10 min. The amplified PCR fragments were then sent to a private company for sequencing (Shanghai Sangon Biological Engineering Technology and Service Co., Ltd., China).

### ﻿Alignment and phylogenetic analyses

Sequence contigs of SSU, LSU, ITS, *tef1-α* and *rpb2* gene regions were assembled, trimmed, and manually checked using BioEdit v. 7.0.5.3 (Hall 1999). The consensus sequences generated in this study were supplemented by additional sequences obtained from GenBank (Table 1) based on BLAST searchers and the past literature (Wanasinghe et al. 2018; Pem et al. 2019; Hongsanan et al. 2020; Hyde et al. 2021; Tennakoon et al. 2021). Multiple sequence alignments with individual gene datasets were generated with MAFFT v.7. online platform (Katoh et al. 2019) and trimmed with TrimAl v. 1.3 (Capella-Gutiérrez et al. 2009) via the web server Phylemon2 (http://phylemon.bioinfo.cipf.es/utilities.html; accessed on 1 January 2023). Individual datasets were concatenated into a combined dataset using BioEdit v. 7.0.5.3. The individual and combined datasets were subjected to maximum likelihood (ML) and Bayesian (BI) phylogenetic inference.

**Table 1.** GenBank accession numbers of the strains used for phylogenetic analysis in this study. “^#^” Denotes ex-type, ex-isotype, ex-paratype or ex-epitype strains. “†’ Denotes type species. Newly generated sequences are shown in bold. NA: sequence data is not available.

Species	Strain no	GenBank accession no.				
		ITS	LSU	SSU	*tef*1-α	*rpb*2
*Alpinariarhododendri* ^†^	KT 2520	LC203335	LC203360	LC203314	LC203388	LC203416
*Alpinariarhododendri* ^†^	CBS 141994^#^	KY189973	KY189973	KY190004	KY190009	KY189989
*Aposphaeriacorallinolutea*	MFLU 15-2752	KY554202	KY554197	KY554200	KY554205	KY554207
*Aposphaeriacorallinolutea*	MFLU 16-2412	MT177916	MT177943	MT177971	NA	MT432199
*Bertiellaellipsoidea*	MFLUCC 17-2015	MG543922	MG543913	NA	MG547226	MG547224
*Bertiellafici*	NCYU 19-0073^#^	NA	MW063224	MW079352	MW183787	NA
*Beverwykellapulmonaria* ^†^	CBS 283.53^#^	KY189974	KY189974	KY190005	NA	KY189990
*Byssosphaeriamacarangae*	MFLUCC 17-2655^#^	MH389782	MH389778	MH389780	MH389784	NA
*Byssosphaeriataiwanense*	MFLUCC 17-2643^#^	MH389783	MH389779	MH389781	MH389785	NA
*Camposporiumdulciaquae*	MFLU 21-0015^#^	MT864352	MT860430	MW485612	MW537104	NA
*Camposporiumseptatum*	MFLUCC 19-0483^#^	MN758892	MN759023	MN758958	MN784096	MT023017
*Cyclothyriellarubronotata* ^†^	CBS 121892	KX650541	KX650541	NA	KX650516	KX650571
*Cyclothyriellarubronotata* ^†^	CBS 141486^#^	KX650544	KX650544	KX650507	KX650519	KX650574
***Dematiomelanommayunnanense*** ^†^	**KUNCC 23-12728** ^#^	**OQ225528**	**OQ360647**	**OQ360651**	**OQ413238**	**OQ413234**
***Dematiomelanommayunnanense*** ^†^	**KUNCC 23-12730**	**OQ225529**	**OQ360648**	**OQ360652**	**OQ413239**	**OQ413236**
***Dematiomelanommayunnanense*** ^†^	**CGMCC 3.23744**	**OQ225530**	**OQ360649**	**OQ360653**	**OQ413240**	**OQ413237**
***Dematiomelanommayunnanense*** ^†^	**KUNCC 22-12677**	**OQ225531**	**OQ360650**	**OQ360654**	**OQ413241**	**OQ413235**
*Fusiconidiummackenziei* ^†^	MFLUCC 14-0434^#^	NA	KX611112	KX611114	KX611118	KX611116
*Gemmamycespiceae*	CBS 141759^#^	KY189977	KY189977	NA	KY190012	KY189993
*Gemmamycespiceae*	CBS 141555	KY189976	KY189976	KY190006	KY190011	KY189992
*Herpotrichiajuniperi*	CBS 200.31	NA	DQ678080	DQ678029	DQ677925	DQ677978
*Herpotrichiamacrotricha*	GKM 196N	NA	GU385176	NA	GU327755	NA
*Herpotrichiaxiaokongense*	KUMCC 21-0004^#^	NA	MZ408889	MZ408891	MZ394066	NA
*Marjiatianshanica* ^†^	TASM 6121^#^	MG828910	MG829020	MG829127	MG829207	NA
*Marjiauzbekistanica*	TASM 6122^#^	MG828911	MG829021	MG829128	MG829208	NA
*Melanocamarosporiumgaliicola* ^†^	MFLUCC 13-0545^#^	NA	OR206417	OR206407	NA	NA
*Melanocamarosporioidesugamica* ^†^	MFLU 17-0064^#^	MH000192	MH000190	MH000191	MH006610	NA
*Melanocucurbitariauzbekistanica* ^†^	MFLUCC 17-0829^#^	MG828912	MG829022	MG829129	MG829209	NA
*Melanodiplodiatianschanica* ^†^	MFLUCC 17-0805^#^	MG828913	MG829023	MG829130	MG829210	MG829256
*Melanodiplodiatianschanica* ^†^	TASM 6111^#^	MG828914	MG829024	MG829131	MG829211	NA
*Melanodiplodiatianschanica* ^†^	TASM 6112	MG828915	MG829025	MG829132	MG829212	MG829257
*Melanommajaponicum*	MAFF 239634^#^	LC203321	LC203339	LC203293	LC203367	LC203395
*Melanommajaponicum*	KT 3425^#^	LC203320	LC203338	LC203292	LC203366	LC203394
*Melanommapulvis-pyrius* ^†^	CBS 124080^#^	MH863349	GU456323	GU456302	GU456265	GU456350
*Monoseptellarosae* ^†^	MFLUCC 17-0815^#^	MG828916	MG829026	MG829133	MG829213	NA
*Muriformistrickeriarosae*	MFLU 16-0227^#^	MG828918	MG829028	MG829135	MG829215	NA
*Muriformistrickeriarubi* ^†^	MFLUCC 17-2550	MG828919	MG829029	MG829136	MG829216	NA
*Muriformistrickeriarubi* ^†^	MFLUCC 15-0681^#^	NA	KT934253	KT934257	KT934261	NA
*Neobyssosphaeriaclematidis* ^†^	MFLUCC 17-0794^#^	NA	MT214566	MT408594	NA	NA
*Petrakiaechinata* ^†^	WU 36922	KY189980	KY189980	KY190007	KY190015	KY189996
*Petrakiaechinata* ^†^	CBS 133070	JQ691628	LC203352	LC203306	LC203380	LC203408
*Phragmocephalaatra*	MFLUCC 15-0021	KP698721	KP698725	KP698729	NA	NA
*Phragmotrichumchailletii* ^†^	CPC 33263^#^	MN313812	MN317293	NA	MN313858	MN313840
*Phragmotrichumchailletii* ^†^	CPC 33341	MN313813	MN317294	NA	MN313859	MN313841
*Phragmocephalagarethjonesii*	MFLUCC 15-0018^#^	KP698722	KP698726	KP698730	NA	NA
*Pleotrichocladiumopacum* ^†^	AU-BD04	JN995638	JN941370	JN938733	NA	NA
*Pleotrichocladiumopacum* ^†^	FMR 12416^#^	KY853462	KY853523	NA	NA	NA
*Praetumpfiaobducens* ^†^	WU 36895	KY189982	KY189982	NA	KY190017	KY189998
*Praetumpfía obducetis* ^†^	CBS 141474^#^	KY189984	KY189984	KY190008	KY190019	KY190000
*Pseudobyssosphaeriabambusae* ^†^	MFLU 18-0151^#^	MG737556	MG737555	NA	MG737557	NA
*Pseudostrickeriaononidis*	MFLUCC 14-0949^#^	NA	KT934255	KT934259	KT934263	KT934264
*Pseudostrickeriarosae*	MFLUCC 17-0643^#^	MG828954	MG829065	MG829169	MG829234	NA
*Pseudotrichiamutabilis*	SMH 1541	NA	GU385209	NA	NA	NA
*Pseudotrichiamutabilis*	WU 36923	KY189988	KY189988	NA	KY190022	KY190003
*Sarimanaspseudofluviatile*	KT760^#^	LC001717	LC001714	LC001711	NA	NA
*Sarimanasshirakamiense* ^†^	HHUF 30454^#^	NR_138017	NG_059803	NG_061263	NA	NA
*Seifertiaalpina*	ZT Myc 59953^#^	MK502003	MK502026	MK502037	MK502083	MK502059
*Seifertiaazaleae* ^†^	ZT Myc 59954	MK502004	MK502028	MK502038	MK502085	MK502061
*Tumulariaaquatica*	CBS 212.46^#^	MH856165	MH867689	NA	NA	NA
*Tumulariatuberculata* ^†^	CBS 256.84	NA	GU301851	NA	GU349006	NA
*Uzbekistanicarosae-hissaricae* ^†^	MFLUCC 17-0819^#^	MG828975	MG829087	MG829187	MG829242	MG829262
*Uzbekistanicayakutkhanika*	MFLUCC 17-0842^#^	MG828978	MG829090	MG829190	MG829245	MG829265

AU-BD: Personal collection of Gareth Griffith; CBS: Culture Collection of the Westerdijk Fungal Biodiversity Institute, Netherlands; CPC: Personal collection of P.W. Crous, Netherlands; FMR: culture collection of the Faculty of Medicine at the Rovira i Virgili University, Spain; GKM: Personal collection of George K. Mugambi; HHUF: Herbarium of Hirosaki University, Fungi, Japan; KT: Personal collection of Kazuaki Tanaka; KUNCC: Kunming Institute of Botany Culture Collection, China; MAFF: Genebank Project of NARO, Japan; MFLUCC/MFLU: Mae Fah Luang University Culture Collection, Chiang Rai, Thailand; NCYU: National Chiayi University Herbarium, Taiwan, China; NFCCI: National Fungal Culture Collection of India; SMH: Personal collection of Sabine M. Huhndorf; TASM: Tashkent Mycological Herbarium of the Institute of Botany, Uzbekistan; ZT Myc: Fungal collection of the ETH (Eidgenössische Technische Hochschule) Zurich, Switzerland.

The FASTA format of the combined datasets was converted to PHYLIP format via the Alignment Transformation Environment (ALTER) online program (http://www.sing-group.org/ALTER/; accessed on 1 January 2023) and used for maximum likelihood analysis (ML). Maximum likelihood trees were inferred using RAxML-HPC2 on the XSEDE (8.2.12) (Stamatakis 2014) in CIPRES Science Gateway v.3.3 (Miller et al. 2010) online platform using the GTR+GAMMA model of nucleotide evolution with 1000 bootstrap replicates. The alignments containing SSU, LSU, ITS, *tef1-α* and *rpb2* were converted to NEXUS format (.nxs) using CLUSTAL X (2.0) and PAUP v. 4.0b10 (Thompson et al. 1997; Swofford 2002). The evolutionary models for BI analysis were selected independently for each locus using MrModeltest v. 2.3 (Nylander et al. 2008) under the Akaike Information Criterion (AIC). GTR+I+G was selected as the best-fit model for all five analyses and processed for Bayesian inference analysis (BI). BI analysis was conducted using MrBayes on XSEDE (3.2.7a) (Ronquist et al. 2012) in CIPRES Science Gateway v.3.3 setting GTR+I+G, six simultaneous Markov chains were run for 50,000,000 generations, and the trees were sampled for every 100^th^ generation. The first 25% of trees were considered burn-in and discarded. The two runs were considered converged when the standard deviation of split frequencies dropped below 0.01.

The Fig. Tree v 1.4.0 program (Rambaut 2012) was used to visualize the phylogenetic trees and reorganized in Microsoft PowerPoint before being saved in PDF format and finally converted to TIFF format using Adobe Photoshop CS6 Extended version 13.0.1 (Adobe Systems, CA, USA).

In this paper, we follow the guidelines of Aime et al. (2021), Chethana et al. (2021) and Pem et al. (2021) when introducing new species.

## ﻿Results

### ﻿Phylogenetic analysis

**Figure 1.**
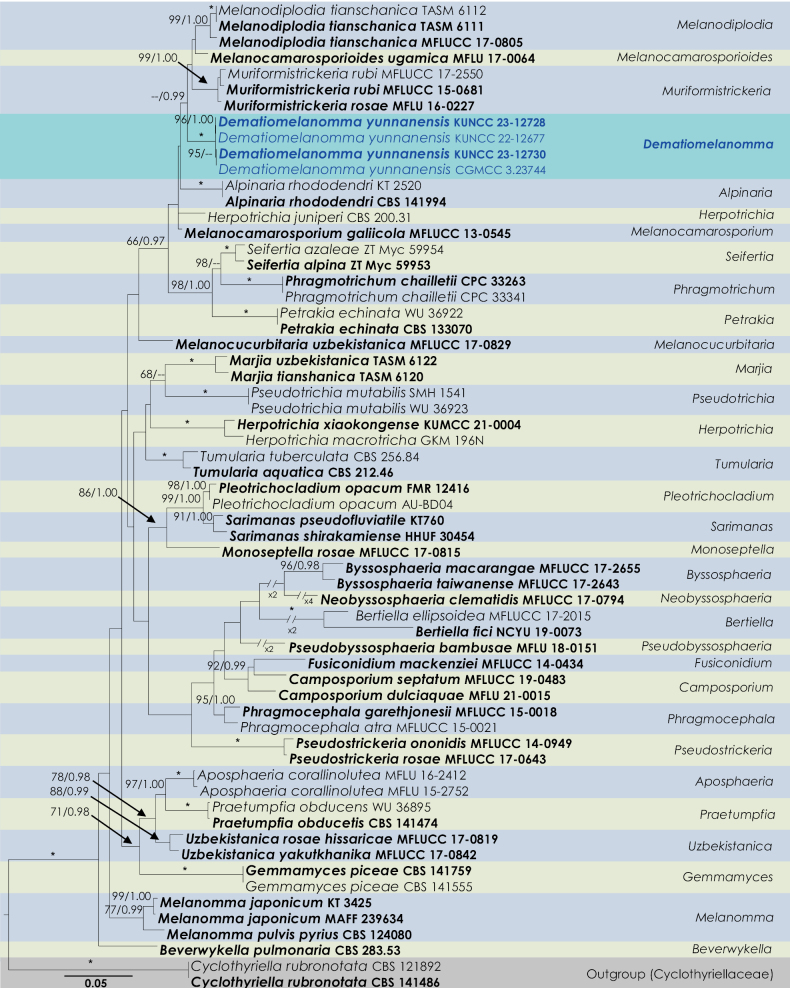
Maximum likelihood (ML) tree resulting from a RAxML analysis of the combined (SSU, LSU, ITS, *tef*1-α and *rpb*2) alignment of the analyzed genera in Melanommataceae. The tree is rooted with *Cyclothyriellarubronotata* (CBS 121892 and CBS 141486). Bootstrap support values for ML equal to or greater than 70% and the Bayesian posterior probabilities equal to or higher than 0.95 PP are indicated above the nodes as ML/PP. Branches with an asterisk (*) indicate ML = 100% and PP = 1.00. Ex-type, ex-isotype, ex-paratype or ex-epitype strains are in bold, and the new isolate is indicated in blue.

The combined sequence data of SSU, LSU, ITS, *tef*1-α and *rpb*2 comprised 62 strains of Melanommataceae and *Cyclothyriellarubronotata* (CBS 121892 and CBS 141486) as outgroup taxa (Fig. 1). A total of 4,678 characters, including gaps, were obtained in the phylogenetic analysis, viz. SSU = 1–1,020 bp, LSU = 1,021–1,867 bp, ITS = 1,868–2,398 bp, *tef1-α* = 2,399–3,828 bp, *rpb2* = 3,829–4,678 bp. The RAxML analysis of the combined dataset yielded a best scoring tree with a final ML optimization likelihood value of -25464.925021. The matrix had 1513 distinct alignment patterns, with 30.36% undetermined characters or gaps. Parameters for the GTR + I + G model of the combined amplicons were as follows: Estimated base frequencies; A = 0.244284, C = 0.245141, G = 0.266746, T = 0.243829; substitution rates AC = 1.669345, AG = 5.027956, AT = 1.689378, CG = 1.259972, CT = 11.771779, GT = 1.000; proportion of invariable sites I = 0.573815; and gamma distribution shape parameter α = 0.523908. The Bayesian analysis ran 1161000 generations before the average standard deviation for split frequencies reached below 0.01 (0.009966). The analyses generated 11611 trees from which 8709 were sampled after 25% of the trees were discarded as burn-in. The alignment contained a total of 1516 unique site patterns. The ML and BI analyses showed similar tree topologies and were congruent. The clade and genera arrangement in the present study agrees with Tennakoon et al. (2021).

Four strains of our new species, *Dematiomelanommayunnanense* (KUNCC 22-12677, CGMCC 3.23744, KUNCC 23-12728 and KUNCC 23-12730), nested as a monophyletic clade with 100% ML and 1.00 PP support values (Fig. 1). This clade has a sister affiliation to *Muriformistrickeriarubi*, *Muriformistrickeriarosae*, *Melanocamarosporioidesugamica* and *Melanodiplodiatianschanica* in Melanommataceae. Besides establishing a new genus, our multi-gene phylogeny also clarifies intergeneric relationships within Melanommataceae. In particular, we note that all the genera (except *Camposporium*) herein are monophyletic lineages.

### ﻿Taxonomy

#### ﻿ Dematiomelanomma

Taxon classificationFungiPleosporalesMelanommataceae

Wanas., Y. Gao, H. Gui & K.D. Hyde gen. nov.

72439201-AAA6-56EA-9947-413C9D01145E

 848034

Facesoffungi Number: FoF14046

##### Etymology.

The generic epithet comes from combining the words *Dematio* and *Melanomma*, meaning brown spores in Melanommataceae.

##### Description.

Saprobic on dead woody stalks. ***Sexual morph***: Ascomata solitary or gregarious, superficial, black, globose to subglobose, ostiolate. Ostiole central, papillate or apapillate, filled with hyaline cells. Peridium multi-layered, comprising cells of textura angularis. Hamathecium comprising of hyaline, filamentous, branched or unbranched, septate pseudoparaphyses. Asci eight-spored, bitunicate, fissitunicate, cylindrical to cylindric-clavate, with a pedicel, rounded and thick-walled at apex, with an ocular chamber. Ascospores uniseriate, sometimes overlapping, muriform, ellipsoidal to fusiform, narrowly rounded at ends, initially hyaline, becoming brown at maturity, with transverse septum appearing first, later becoming vertically septate, smooth-walled, with a mucilaginous sheath. ***Asexual morph***: Synanamorphic. Conidiomata pycnidial, solitary or gregarious, mostly superficial, obpyriform, dark brown to black, ostiolate. Ostiole single, circular, centrally papillate with periphyses. Conidiomatal wall multi-layered, thick-walled, dark brown, composed of cells of textura angularis, inner layer with hyaline cells. Macroconidiogenous cells enteroblastic, annellidic, integrated, indeterminate, doliiform, smooth-walled, hyaline, arising from the innermost layer of pycnidial wall. Macroconidia medium brown to dark brown, ellipsoidal to fusiform, phragmosporous to muriform, curved to straight. Microconidiogenous cells present or absent in cultures; when present, hyaline, integrated, enteroblastic, percurrently annellidic, ampulliform to subcylindrical. Microconidia present or absent; when present, hyaline, round to oblong or ellipsoidal, with small guttules.

##### Type species.

*Dematiomelanommayunnanense* Y. Gao, Wanas., H. Gui & K.D. Hyde.

#### ﻿ Dematiomelanomma yunnanense

Taxon classificationFungiPleosporalesMelanommataceae

Y. Gao, Wanas., H. Gui & K.D. Hyde sp. nov.

97A543CA-EC48-5708-8A81-263B1AACDF84

 848038

Facesoffungi Number: FoF14016

Figs 2 , 3

##### Etymology.

The specific epithet “yunnanense” refers to Yunnan Province, where the holotype was collected.

##### Holotype.

HKAS 124666.

##### Description.

Saprobic on decaying stalk of *Rubusparvifolius* and *Hypericummonogynum*. ***Sexual morph***: Ascomata 360–440 μm high × 425–500 μm diam. (x̄ = 396 × 460 μm, n = 10), mostly gregarious, black, globose to subglobose, superficial, ostiolate. Ostiole central, minute papillate, filled with hyaline cells. Peridium 30–60 μm thick (x̄ = 47 μm, n = 30), irregularly multi-layered, comprising brown to black cells of textura angularis, with inner layer composed of flattened, hyaline cells of textura angularis. Hamathecium composed of 1–2.5 μm (x̄ = 1.7 μm, n = 30) wide, septate, hyaline, branched pseudoparaphyses. Asci (165–)180–223(–232) × (18–)19–25(–26) μm (x̄ = 200 × 22 μm, n = 20, SD = 22 × 3.3), eight-spored, bitunicate, fissitunicate, cylindrical, pedicellate, apically rounded, thick-walled at apex, with a minute ocular chamber. Ascospores (27–)29–33(–34) × (9–)10.2–12.6(–14.5) μm (x̄ = 30.8 × 11.4 μm, n = 30, SD = 2 × 1.2), muriform, with 3–7 transverse septa, and 1–3 vertical septa, with transverse septum appearing first, then vertical septa gradually emerge, mostly ellipsoidal or fusiform, rounded at both ends, initially hyaline, becoming dark brown at maturity, constricted at septa, smooth-walled, with a mucilaginous sheath. ***Asexual morph***: Conidiomata 240–360 μm high × 185–245 µm diam (x̄ = 279 × 214 μm, n = 10), pycnidial, solitary or gregarious, superficial, obpyriform, dark brown to black, ostiolate. Ostiole 122–134 μm high × 57–62 µm wide (x̄ = 125 × 60 μm, n = 5), single, centric, circular, with hyaline periphyses, ostiolate, e single, circular, centrally papillate with or without periphyses. Conidiomatal wall multi-layered, 30–50 µm wide (x̄ = 34 μm, n = 30), composed of brown cells of textura angularis, with inner layer comprising hyaline cells. Macroconidiogenous cells (5–)5.5–8.7(–9.7) × (4–)5.8–8(–9.5) μm (x̄ = 7 × 7 μm, SD = 1.6 × 1.3 μm, n = 20), enteroblastic, annellidic, integrated, indeterminate, doliiform, smooth-walled, hyaline, arising from the inner wall cells of pycnidial wall. Macroconidia (30–)32.5–37.5(–39) × (8–)10–12(–14) μm (x̄ = 35 × 11 μm, SD = 2.5 × 1.2, n = 30), medium brown to dark brown, ellipsoidal to fusiform, phragmosporous to muriform, with 6–9 transverse septa, and 1–2 longitudinal septa, 1–2 oblique septa, curved to straight.

**Figure 2.**
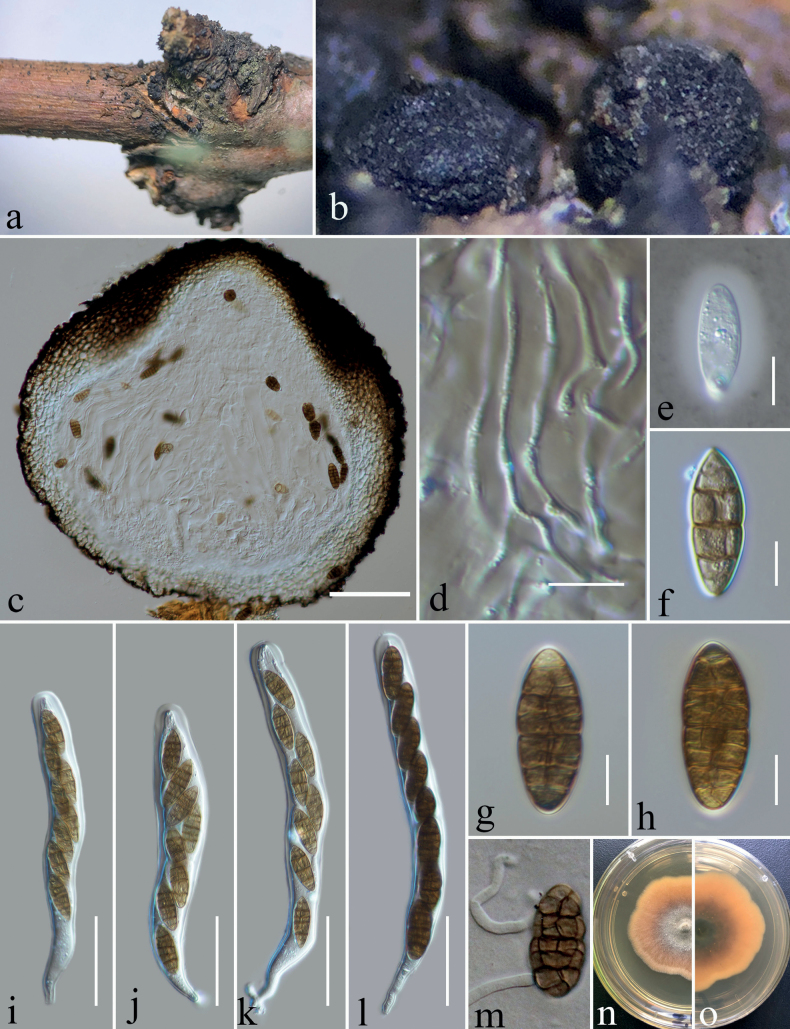
Sexual morph of *Dematiomelanommayunnanense* (HKAS 124667) on decaying stalk of *Rubusparvifolius* L. **a, b** ascomata in face view **c** vertical section of the ascoma **d** pseudoparaphyses **e** an ascospore in Indian Ink to show a sheath **f–h** ascospores **i–l** Asci **m** germinating ascospore **n, o** surface and reverse of colony on PDA. Scale bars: 100 μm (**c**); 10 μm (**d–h**); 50 μm (**i–l**).

**Figure 3.**
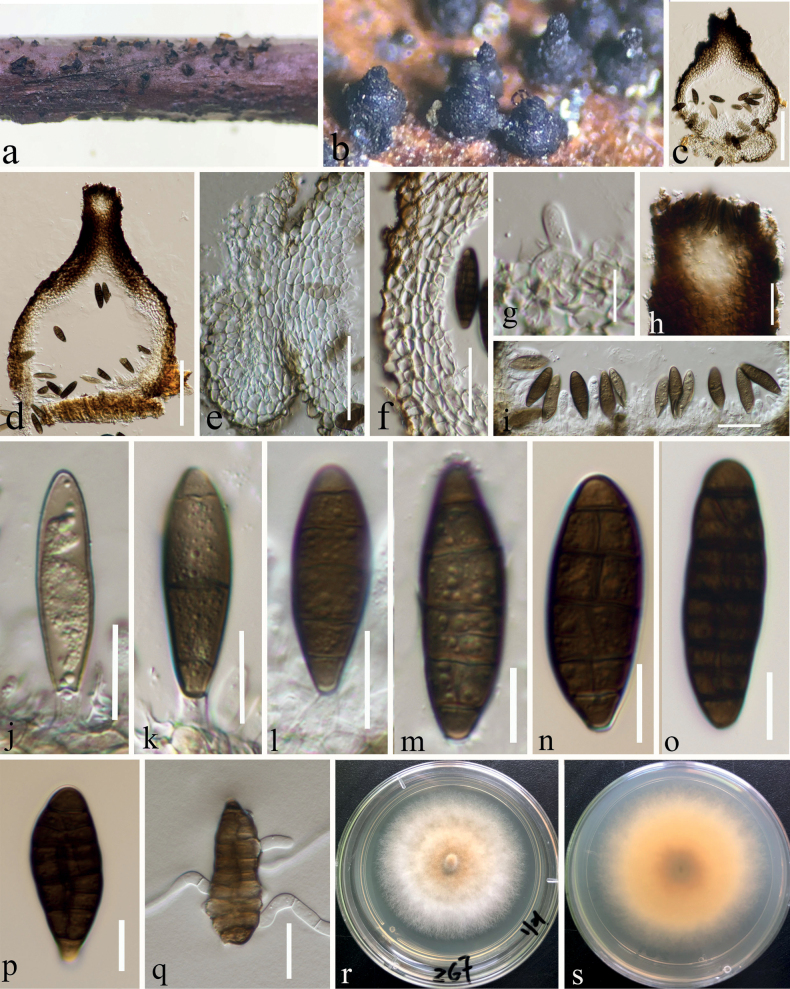
Asexual morph of *Dematiomelanommayunnanense* on a dead stalk of *Hypericummonogynum* L. (HKAS 124666, holotype) **a, b** conidiomata in face view **c, d** vertical section of conidiomata **e** vertical section of the base of the pulvinate-structure **f** conidioma wall **g** conidiogenous cells arising from the wall and developing conidia **h** vertical section through ostiole **i** developing stages of conidia **j–p** conidia **q** geminating conidia **r** cultures on PDA from above **s** cultures on PDA from reverse. Scale bars: 100 μm (**c, d**); 50 μm (**e**); 30 μm (**f**); 10 μm (**g**); 20 μm (**h**); 30 μm (**i**); 15 μm (**j–l**); 10 μm (**m–p**); 20 μm (**q**).

##### Culture characteristics.

**Figure 4.**
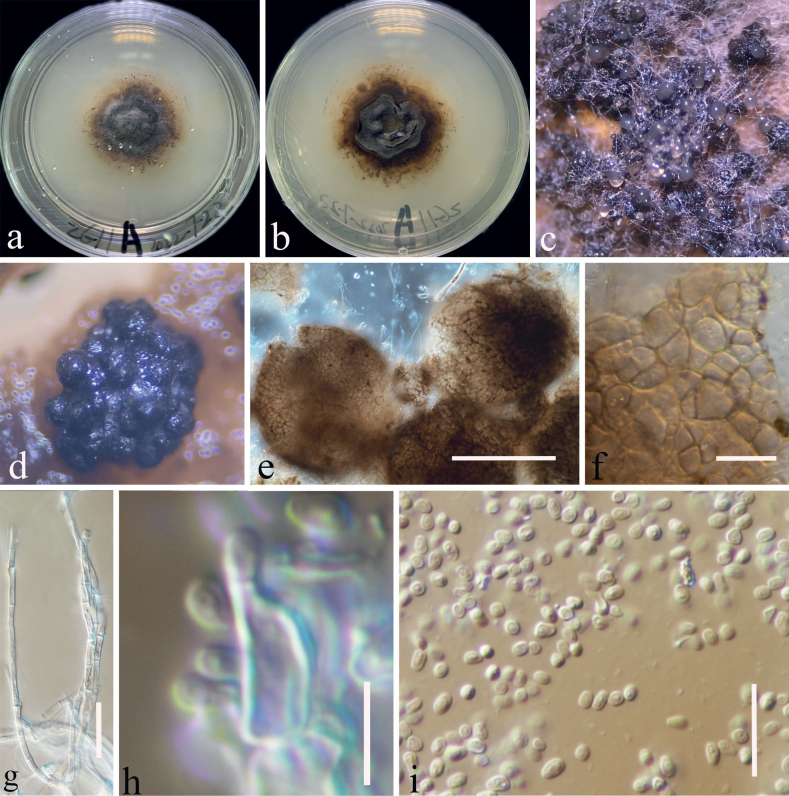
Asexual morph of *Dematiomelanommayunnanense* from the culture (CGMCC 3.23744) on PDA **a, b** colony of the sexual morphic stage after 24 weeks on PDA (**b** from the bottom) **c–e** conidiomata **f** conidioma wall **g** mycelium **h** conidiogenous cells arising from the wall and developing conidia **i** conidia. Scale bars: 100 μm (**e**); 15 μm (**f**); 30 μm (**g**); 5 μm (**h**); 15 μm (**i**).

Ascospores germinated on PDA within 20 hours, and germ tube initially produced from the 2 ends of the ascospores. Colonies on PDA reaching 25 mm in 3 weeks at room temperature (25–27 °C), irregular, center is slightly raised, panniform, mycelium grows on the surface of PDA, brown from the above, brown in the center gradually becoming yellow towards the edges from the below. Conidia germinating on PDA within 24 hours. Colonies on PDA reaching 20 mm in 2 weeks at 25–27 °C, circular, slightly raised, floccose, white from the above and yellowish from the center and below, smooth with filamentous edge. Mycelium 2–3 μm broad, (x̄ = 2.5 μm, n = 30), septate, hyaline, branched and sporulated after 24 weeks. Asexual morph on PDA (Fig. 4): Conidiomata 60–155 μm high × 62–145 µm diam (x̄ = 123 × 119 μm, n = 10), pycnidial, gregarious, immersed to superficial, globose to subglobose, dark brown to black, ostiolate, with clear gelatinous substance at the top. Peridium thin, composed of brown cells of textura angularis to globulosa. Microconidiogenous cells (4.5–)6.5–8.6(–9) × (2.5–)3.5–6(–6.5) μm (x̄ = 7.5 × 4.7 μm, SD = 1.1 × 1.2 μm, n = 25), hyaline, integrated, enteroblastic, percurrently annellidic, ampulliform to subcylindrical. Microconidia (2.5–)2.7–3.6(–5) × (1.6–)1.8–2.2(–2.5) μm (x̄ = 3.2 × 2 μm, SD = 0.44 × 0.2 μm, n = 30), hyaline, aseptate, round to oblong or ellipsoidal, with small guttules.

##### Material examined.

China, Yunnan Province, Zhaotong city, Daguan County Grassland (27°44'23"N, 103°47'59"E), on decaying stalk of *Hypericummonogynum*, 21 August 2021, ZG7FB (HKAS 124666, holotype, asexual morph), ex-type, KUNCC 23-12728. *ibid*., ZG7 (HKAS 127122, isotype), ex-isotype, KUNCC 22-12677. China, Yunnan Province, Zhaotong city, Daguan County Grassland (27°44'23"N, 103°47'59"E), on decaying stalk of *Rubusparvifolius*, 21 August 2021, Ying Gao, ZG11FB (HKAS 124667, sexual morph), living culture, KUNCC 23-12730. *ibid*., ZG11 (HKAS 127123), living culture, CGMCC 3.23744.

##### Note.

Four strains of *Dematiomelanomma* clustered in Melanommataceae as a strongly supported monophyletic clade (Fig. 1) in both ML and BI of a concatenated SSU, LSU, ITS, *tef*1-α and *rpb*2 dataset. Two specimens belong to the sexual morph (KUNCC23-12730, CGMCC 3.23744) collected on decaying stalks of *Rubusparvifolius* and two asexual morphic coelomycetous fungi (KUNCC 23-12728, KUNCC 22-12677) were collected on the decaying stem of *Hypericummonogynum* from grassland in Zhaotong, Yunnan. There was no significant difference between the morphological characteristics of these sexual morphic specimens or asexual morphic specimens and DNA-based sequence comparisons of these collections. Therefore, we introduce them as different collections of *Dematiomelanommayunnanense* sp. nov.

## ﻿Discussion

In this study, we described and illustrated a new species in a new genus of microfungi, Dematiomelanommayunnanense from dead stalks of *Hypericummonogynum* and *Rubusparvifolius* from Zhaotong, Yunnan, based on morphological and molecular analyses (Figs 1–4). *Dematiomelanommayunnanense* is introduced with both asexual and sexual morphological features. Pleomorphy, the variation in morphology and structure among different taxa, is a common characteristic of several fungi (Rossman et al. 2015). This variability can be observed in various characteristics such as color, shape, and size of the fruiting body, as well as in the conidial and ascospore structures. Two levels of pleomorphy, teleomorphosis-anamorphosis and pleoanamorphy (synanamorphs), can be observed in fungi (Rogerson 1988). Data on teleomorph-anamorph connections and pleoanamorph connections, together with the analysis of conidium ontogeny, are important considerations in the taxonomy of Ascomycota. In recent years, knowledge regarding pleomorphy and its dramatic examples has increased significantly (Rossman et al. 2016). The family Melanommataceae is known for its pleomorphism, particularly in morphology and structure among teleomorph-anamorph connections. For instance, *Exosporiellafungorum* has brown, fusiform 1-septate ascospores and 4 transversely septate, brown, oblong conidia (Tian et al. 2015). *Pseudostrickeriaononidis* has ellipsoidal, brown, muriform ascospores, while their conidia are aseptate, brown, and globose to subglobose (Tian et al. 2015). *Gemmamycespiceae* has broadly ellipsoid, brown muriform ascospores, and vermiform, hyaline conidia with 7–33 septa (Jaklitsch and Voglmayr 2017). *Praetumpfiaobducens* has ellipsoidal, muriform pigmented ascospores in the sexual morph and oblong to cylindrical, 1-celled, hyaline conidia in the asexual morph (Jaklitsch and Voglmayr 2017). *Pseudodidymellafagi* has fusiform, 1-septate, hyaline ascospores and pyrenochaeta-like, hyaline, ellipsoidal conidia (Hashimoto et al. 2017). *Uzbekistanicarosae-hissaricae* has ellipsoidal, brown, muriform ascospores, and *U.yakutkhanika* has 1-septate, oval to ovoid conidia (Wanasinghe et al. 2018). *Muriformistrickeriarubi* has ellipsoidal, muriform, brown ascospores and hyaline, unicellular conidia (Tian et al. 2015). Interestingly, even in the sexual morph within the genus of *Muriformistrickeria*, pleomorphism can be observed, with *M.rosae* having hyaline ascospores while *M.rubi* has pigmented ascospores at maturity (Tian et al. 2015; Wanasinghe et al. 2018). The pleomorphism observed in the family Melanommataceae highlights the diversity of this group of fungi and emphasizes the importance of careful taxonomic identification based on morphological and molecular characteristics.

The asexual morph of this new fungus produces both macro- and micro-conidia in their life cycle (synanamorphs). A quick sporulation using minimal nutrient requirements helps the fungi to escape from unfavorable conditions quickly. Therefore, producing asexual spores (conidia) is beneficial for a fungus, especially to survive under adverse environmental conditions via the dispersal of a sufficient number of spores to many potentially viable sites. The species in Ascomycota produce several types of asexual spores, such as macroconidia, microconidia, and chlamydospores. Some species, such as *Neurosporacrassa* have variations even among the microconidia, *i.e.* blastoconidia, arthroconidia through micro-conidiogenesis (Maheshwari 1991). However, the production of microconidia is normally suppressed in most of the Ascomycota. It is evident that microconidia should provide some advantages to the life cycle of the fungal species capable of producing them. For example, microconidia produced by *Metarhiziumacridum* are more thermo tolerant than typical aerial conidia (Zhang et al. 2010). The retention of microconidia development indicates biological meaning in nature (Jung et al. 2014). Therefore, it is important to understand this process in the evolutionary context.

The sexual morph of *Dematiomelanomma* morphologically resembles the genera such as *Gemmamyces*, *Marjia*, *Melanocucurbitaria*, *Muriformistrickeria*, *Praetumpfia*, *Pseudostrickeria* and *Uzbekistanica* in having muriform ascospores in Melanommataceae (Wanasinghe et al. 2018). Although there is some morphological overlap between *Dematiomelanomma* and the genera mentioned above, except *Muriformistrickeria* (Table 2), they are not closely associated in the phylogenetic analyses. In the phylogenetic analyses, *Dematiomelanomma* is monophyletic with *Melanocamarosporioides*, *Melanodiplodia* and *Muriformistrickeria* (Fig. 1). However, their macroconidia are different. *Melanocamarosporioides* has camarosporium-like conidia (Pem et al. 2019), *Melanodiplodia* has diplodia-like conidia (Wanasinghe et al. 2018), and *Muriformistrickeria* has phoma-like conidia (Tian et al. 2015) whereas *Dematiomelanomma* produces camarographium-like conidia (Wijayawardene et al. 2016). Furthermore, the sexual morph of the *Dematiomelanomma* and *Muriformistrickeria* are different in their asci and ascospore characteristics (Table 3). Most sexual genera of Melanommataceae have trabeculae which are narrow, frequently anastomosing pseudoparaphyses which are embedded in a gelatinous matrix (Liew et al. 2000). In the case of *Dematiomelanomma* the pseudoparaphyses are similar to trabeculae but differ in having swollen regions.

**Table 2.** Synopsis of sexual morphic features of the phylogenetically closely related species to *Dematiomelanommayunnanense*.

Species	Ascomata	Asci	Ascospores		Reference
			Shape	Septa	
*Dematiomelanommayunnanense*	Globose to subglobose, black, minute papillate.	Fissitunicate, cylindrical, pedicellate, apically rounded, thick-walled at the apex, with a minute ocular chamber.	Muriform, mostly ellipsoidal or fusiform, narrowly rounded at the ends, initially hyaline, becoming dark brown at maturity, smooth-walled, with a mucilaginous sheath.	3–7 transversely septate, and 1–3 vertical septa.	This study
*Muriformistrickeriarubi*	Globose or flattened, semi-immersed to erumpent, dark brown to black, coriaceous, smooth, ostiolate.	Fissitunicate, cylindrical to cylindric-clavate, short pedicellate apically rounded, with an ocular chamber.	Ellipsoidal, muriform, initially light yellow, becoming yellowish-brown at maturity, conical and narrowly rounded at the ends, lower cell narrows and longer, smooth-walled, with a thick mucilaginous sheath.	4–6 transversely septate, with 2–4 vertical septa.	Tian et al. (2015)
*Muriformistrickeriarosae*	Broadly oblong and flattened, dark brown to black, coriaceous, ostiolate.	Fissitunicate, cylindrical to cylindric-clavate, pedicellate, thick-walled at the apex, with minute ocular chamber.	Overlapping 1–2-seriate, muriform, ellipsoidal to subfusiform, slightly curved, upper part wider than the lower part, hyaline, with rounded ends, without a mucilaginous sheath.	3–4-transversely septate, with 1 vertical septa.	Wanasinghe et al. (2018)

**Table 3.** Synopsis of asexual morphic features of the phylogenetically closely related species to *Dematiomelanommayunnanense*.

Species	Conidiomata	Conidiogenous cells	Conidia		Reference
			Shape	Septa	
*Dematiomelanommayunnanense*	Solitary or gregarious, superficial on the host, globose to subglobose, ostiolate.	Subglobose or cylindrical to subcylindrical, hyaline, smooth, arising from conidiomata wall.	Fusiform or long fusiform, mostly straight, infrequently slightly curved, pale brown when young, becoming dark brown at maturity.	4–8 transverse septa, and 1–2 longitudinal septa.	This study
*Dematiomelanommayunnanense*	Gregarious, superficial on PDA, subglobose, ostiolate, clear gelatinous substance at the top.	Urn-shaped and ampuliform, hyaline, smooth.	Short cylindrical, subglobose, hyaline when young, becoming pale brown at maturity.	Aseptate	This study
*Muriformistrickeriarubi*	Mostly solitary, semi-immersed to immersed in the host, globose, ostiolate, apapillate.	Cylindrical to subcylindrical, hyaline, the first conidium produced holoblastically and subsequent conidia enteroblastically forming typical phialides with periclinal thickenings.	Oval to ovoid, widest in the center, apex obtuse, sometimes guttulate when young, initially hyaline, becoming light brown, moderately thick-walled, wall externally smooth, roughened on the inner surface.	Unicellular	Wanasinghe et al. (2018)
*Melanocamarosporioidesugamica*	Scattered, solitary or gregarious, to erumpent, uniloculate, ellipsoidal to subglobose glabrous, ostiolate.	Annelidic, holoblastic, discrete oblong to ampulliform, hyaline to darkbrown, multiseptate, smooth-walled.	Globose, ellipsoidal or ovoid with obtuse ends, hyaline at first, becoming pale brown to dark-brown at maturity, smooth- and thick-walled.	3–4 transverse septa and 1–3 longitudinal septa.	Pem et al. (2019)
*Melanodiplodiatianschanica*	Pycnidial, stromatic, mostly solitary, semi-immersed to immersed, globose, ostiolate, apapillate.	Cylindrical to subcylindrical, hyaline, the first conidium produced holoblastically and subsequent conidia enteroblastically forming typical phialides with periclinal thickenings.	Detached or still attached to conidiogenous cells conidia, hyaline, sepia or blackish brown, moderately thick-walled, wall externally smooth, roughened on the inner surface, oval to ovoid, widest in the center, apex obtuse, sometimes guttulate when young.	Unicellular or 1-septate.	Wanasinghe et al. (2018)

From the available literature, it appears that the macroconidia of *Dematiomelanomma* are similar to those of *Amarenographium*, *Camarographium*, *Myxocyclus*, and *Shearia*. Among the *Amarenographium* species, *Amarenographiumammophilae* (Wijayawardene et al. 2016) and *A.ammophilicola* (Dayarathne et al. 2020) have similar shaped and septate brown conidia to *Dematiomelanomma*, but they are phylogenetically grouped with Phaeosphaeriaceae species. *Camarographiumabietis* (Grove, 1937) among the *Camarographium* species exhibits striking morphological similarities to the new genus, with ellipsoidal to fusiform, muriform, dark-pigmented conidia with oblique septa. However, due to the unavailability of sequence data, the taxonomic placement of this fungus remains unclear. MycoBank database (Crous et al. 2004) currently lists *Myxocycluscenangioides* as the valid name for *Camarographiumabietis*. However, this treatment is not followed by Index Fungorum (2023) or Species Fungorum (2023). The macroconidiogenous cells of *Camarographiumabietis* appear cylindrical and relatively longer (Grove, 1937) than those of *Dematiomelanommayunnanense*, which are short and doliiform. *Myxocycluspolycystis* also exhibits similar conidial morphology to *Dematiomelanomma*, as reported by Tanaka et al. (2005) and Wijayawardene et al. (2016). Saccardo (1908) and Barr (1982) suggested that *Myxocycluspolycystis* might be the asexual morph of *Splanchnonemaargus* based on their co-occurrence on the same host. Later, Tanaka et al. (2005) provided evidence of the congenetic relation of these morphs in culture. Moreover, Vu et al. (2019) provided a putative sequence of the large subunit of *Myxocycluspolycystis* (CBS 222.77: MH872821); however, this sequence did not closely affiliate with Melanommataceae taxa in our primary phylogenetic analyses. Additionally, the acervular conidiomata of *Myxocycluspolycystis* is different from the pycnidial conidiomata of *Dematiomelanomma*. Despite the morphological similarities between the macroconidia of *Shearia* and our new fungus, their phylogenetic affinity is not closely related to Melanommataceae, as reported by Wanasinghe et al. (2020). Species that lack distinctive characteristics for genus-level identification are often collectively deposited in collections as “phoma-like”, resulting in more than 3,000 species epithets being associated with this genus in the MycoBank database (Crous et al. 2004). Therefore, the microconidia of the new genus are too superficial to be compared with existing phoma-like genera.

The vegetation of Zhaotong grassland is composed of 20 plant families, with Asteraceae, Caryophyllaceae, Gramineae, and Rosaceae being the most prevalent (Zhu et al. 2022). However, the ecological significance of *Hypericummonogynum* and *Rubusparvifolius*, and their associations with microorganisms such as fungi, is not well understood. *Hypericummonogynum*, a widely distributed shrub in China’s tropical and subtropical regions, has potential medicinal and ornamental value (Pan et al. 1993; Xi et al. 2007; Zeng et al. 2018; Wu et al. 2021). *Rubusparvifolius*, an important traditional Chinese medicine, is often found in East and South Asia (Roginsky et al. 1996; Yuan et al. 2006). While only six fungal species have been reported from *Hypericummonogynum* (Zhang 2006; Kobayashi 2007), 22 species have been reported from *Rubusparvifolius*, mainly in China and Japan, with a few in Australia, South Korea, Canada, and Russia (Simmonds 1966; Tai 1979; Katumoto 1980; Azbukina 1984; Ginns 1986; Cook and Dubé 1989; Liu and Guo 1998; Cao and Li 1999; Lu et al. 2000; Zhuang 2001; Cho and Shin 2004; Zhuang 2005; Priest 2006; Arzanlou et al. 2007; Kobayashi 2007; Zhuang 2012). In conclusion, the potential ecological and economic significance of *Hypericummonogynum* and *Rubusparvifolius* highlights the need for further research to understand their interactions with fungi in the grasslands of Zhaotong. Wijayawardene et al. (2022b) emphasized the importance of tropical to subtropical regions in discovering novel taxa, particularly with asexual reproduction. This study has identified a new species in a new genus associated with grassland vegetation in Zhaotong, Yunnan, China, suggesting that grasslands in this region have not yet been fully explored and offer opportunities for new fungal discoveries. Therefore, further investigations are required to better understand the fungal diversity and their ecological roles in these grassland ecosystems.

## Supplementary Material

XML Treatment for Dematiomelanomma

XML Treatment for Dematiomelanomma yunnanense

## ﻿Additional information

### Author ORCIDs

Ying Gao https://orcid.org/0000-0001-8671-1978

Tingfang Zhong https://orcid.org/0009-0000-2767-1347

Jayarama D. Bhat https://orcid.org/0000-0002-3800-5910

Antonio Roberto Gomes de Farias https://orcid.org/0000-0003-4768-1547

Turki M. Dawoud https://orcid.org/0000-0002-1444-4185

Kevin D. Hyde https://orcid.org/0000-0002-2191-0762

Weiqiang Xiong https://orcid.org/0009-0002-0210-1625

Yunju Li https://orcid.org/0000-0001-7165-1984

Heng Gui https://orcid.org/0000-0002-0946-1589

Xuefei Yang https://orcid.org/0000-0002-0986-2745

Shixi Wu https://orcid.org/0009-0006-5601-733X

Dhanushka N. Wanasinghe https://orcid.org/0000-0003-1759-3933

### Data availability

All of the data that support the findings of this study are available in the main text.

## References

[B1] Aime MC Miller AN Aoki T Bensch K Cai L Crous PW Hawksworth DL Hyde KD Kirk PM Lücking R May TW Malosso E Redhead SA Rossman AY Stadler M Thines M Yurkov AM Zhang N Schoch CL (2021) How to publish a new fungal species, or name, version 3.0. IMA Fungus 12(1): 11. 10.1186/s43008-021-00063-1 33934723 PMC8091500

[B2] Arzanlou M Groenewald JZ Gams W Braun U Shin HD Crous PW (2007) Phylogenetic and morphotaxonomic revision of *Ramichloridium* and allied genera. Studies in Mycology 58(1): 57–93. 10.3114/sim.2007.58.03 18490996 PMC2104745

[B3] Azbukina ZM (1984) Key to rust fungi of the Soviet Far East. Key to rust fungi of the Soviet Far East, 288 pp.

[B4] Bai Y Cotrufo MF (2022) Grassland soil carbon sequestration: Current understanding, challenges, and solutions. Science 377(6606): 603–608. 10.1126/science.abo2380 35926033

[B5] Bánki O Roskov Y Döring M Ower G Vandepitte L Hobern D Remsen D Schalk P De Walt RE Keping M (2023) Catalogue of Life Checklist (Version 2023-01-12). Catalogue of Life. 10.48580/dfqz

[B6] Bardgett RD Bullock JM Lavorel S Manning P Schaffner U Ostle N Chomel M Durigan G Fry El Johnson D Lavallee JM Provost GL Luo S Png K Sankaran M Hou XY Zhou HK Ma L Ren WK Li XL Ding Y Li YH Shi HX (2021) Combatting global grassland degradation. Nature Reviews Earth & Environment 2(10): 720–735. 10.1038/s43017-021-00207-2

[B7] Barr ME (1982) On the Pleomassariaceae (Pleosporales) in North America. Mycotaxon 15: 349–383.

[B8] Beenken L Gross A Queloz V (2020) Phylogenetic revision of *Petrakia* and *Seifertia* (Melanommataceae, Pleosporales): New and rediscovered species from Europe and North America. Mycological Progress 19(5): 417–440. 10.1007/s11557-020-01567-7

[B9] Buisson E Archibald S Fidelis A Suding KN (2022) Ancient grasslands guide ambitious goals in grassland restoration. Science 377(6606): 594–598. 10.1126/science.abo4605 35926035

[B10] Cao ZM Li ZQ (1999) Rust Fungi of Qinling Mountains. China Forestry Publishing House. China, 188 pp.

[B11] Capella-Gutiérrez S Silla-Martínez JM Gabaldón T (2009) trimAl: A tool for automated alignment trimming in large-scale phylogenetic analyses. Bioinformatics 25(15): 1972–1973. 10.1093/bioinformatics/btp348 19505945 PMC2712344

[B12] Chaiwan N Gomdola D Wang S Monkai J Tibpromma S Doilom M Wanasinghe DN Mortimer PE Lumyong S Hyde KD (2021) An online database providing updated information of microfungi in the Greater Mekong Subregion. Mycosphere 12(1): 1513–1526. 10.5943/mycosphere/12/1/19

[B13] Chen G Yu CQ Shen ZX Li JH (2018) Grassland quality control. Yunnan University Press 313–315.

[B14] Chethana KWT Manawasinghe IS Hurdeal VG Bhunjun CS Appadoo MA Gentekaki E Raspé O Promputtha I Hyde KD (2021) What are fungal species and how to delineate them? Fungal Diversity 109(1): 1–25. 10.1007/s13225-021-00483-9

[B15] Cho WD Shin HD (2004) List of plant diseases in Korea. Korean Society of Plant Pathology. Seoul. Korea, 779 pp.

[B16] Cook RP Dubé AJ (1989) Host-pathogen index of plant diseases in South Australia. South Australian Department of Agriculture, 142 pp.

[B17] Crous PW Gams W Stalpers JA Robert V Stegehuis G (2004) MycoBank: An online initiative to launch mycology into the 21^st^ century. Studies in Mycology 50: 19–22.

[B18] Dayarathne MC Jones EBG Maharachchikumbura SSN Devadatha B Sarma VV Khongphinitbunjong K Chomnunti P Hyde KD (2020) Morpho-molecular characterization of microfungi associated with marine based habitats. Mycosphere 11(1): 1–188. 10.5943/mycosphere/11/1/1

[B19] Ginns JH (1986) Compendium of plant disease and decay fungi in Canada 1960–1980. Canadian Government Publishing Centre 1813: 416. 10.5962/bhl.title.58888

[B20] Grove WB (1937) British Stem- and Leaf-Fungi (Coelomycetes). Cambridge University Press 2: 1–406.

[B21] Hall TA (1999) BioEdit: A user-friendly biological sequence alignment editor and analysis program for Windows 95/98/NT. Nucleic Acids Symposium Series 41(41): 95–98.

[B22] Hashimoto A Matsumura M Hirayama K Fujimoto R Tanaka K (2017) Pseudodidymellaceae fam. nov.: Phylogenetic affiliations of mycopappus-like genera in Dothideomycetes. Studies in Mycology 87(1): 187–206. 10.1016/j.simyco.2017.07.002 PMC5542424 28794574

[B23] Hongsanan S Hyde KD Phookamsak R Wanasinghe DN McKenzie EHC Sarma VV Boonmee S Lücking R Bhat DJ Liu NG Tennakoon DS Pem D Karunarathna A Jiang SH Jones EBG Phillips AJL Manawasinghe IS Tibpromma S Jayasiri SC Sandamali DS Jayawardena RS Wijayawardene NN Ekanayaka AH Jeewon R Lu YZ Dissanayake AJ Zeng XY Luo ZL Tian Q Phukhamsakda C Thambugala KM Dai DQ Chethana KWT Samarakoon MC Ertz D Bao DF Doilom M Liu JK Pérez-Ortega S Suija A Senwanna C Wijesinghe SN Konta S Niranjan M Zhang SN Ariyawansa HA Jiang HB Zhang JF Norphanphoun C de Silva NI Thiyagaraja V Zhang H Bezerra JDP Miranda-González R Aptroot A Kashiwadani H Harishchandra D Sérusiaux E Aluthmuhandiram JVS Abeywickrama PD Devadatha B Wu HX Moon KH Gueidan C Schumm F Bundhun D Mapook A Monkai J Chomnunti P Suetrong S Chaiwan N Dayarathne MC Yang J Rathnayaka AR Bhunjun CS Xu JC Zheng JS Liu G Feng Y Xie N (2020) Refined families of Dothideomycetes: Dothideomycetidae and Pleosporomycetidae. Mycosphere 11(1): 1553–2107. 10.5943/mycosphere/11/1/13

[B24] Hyde KD Gareth Jones EB Liu JK Ariyawansa H Boehm E Boonmee S Braun U Chomnunti P Crous PW Dai DQ Diederich P Dissanayake A Doilom M Doveri F Hongsanan S Jayawardena R Lawrey JD Li YM Liu YX Lücking R Monkai J Muggia L Nelsen MP Pang KL Phookamsak R Senanayake IC Shearer CA Suetrong S Tanaka K Thambugala KM Wijayawardene NN Wikee S Wu HX Zhang Y Aguirre-Hudson B Alias SA Aptroot A Bahkali AH Bezerra JL Bhat DJ Camporesi E Chukeatirote E Gueidan C Hawksworth DL Hirayama K de Hoog S Kang JC Knudsen K Li WJ Li XH Liu ZY Mapook A McKenzie EHC Miller AN Mortimer PE Phillips AJL Raja HA Scheuer C Schumm F Taylor JE Tian Q Tibpromma S Wanasinghe DN Wang Y Xu JC Yacharoen S Yan JY Zhang M (2013) Families of Dothideomycetes. Fungal Diversity 63(1): 1–313. 10.1007/s13225-013-0263-4

[B25] Hyde KD Jeewon R Chen YJ Bhunjun CS Calabon MS Jiang HB Lin CG Norphanphoun C Sysouphanthong P Pem D Tibpromma S Zhang Q Doilom M Jayawardena RS Liu JK Maharachchikumbura SSN Phukhamsakda C Phookamsak R Al-Sadi AM Naritsada Thongklang N Wang Y Gafforov Y Jones EBG Lumyong S (2020) The numbers of fungi: Is the descriptive curve flattening? Fungal Diversity 103(1): 219–271. 10.1007/s13225-020-00458-2

[B26] Hyde KD Suwannarach N Jayawardena RS Manawasinghe IS Liao CF Doilom M Cai L Zhao P Buyck B Phukhamsakda C Su WX Fu YP Li Y Zhao RL He MQ Li JX Tibpromma S Lu L Tang X Kang JC Ren GC Gui H Hofstetter V Ryoo R Antonín V Hurdeal VG Gentikaki E Zhang JY Lu YZ Senanayake IC Yu FM Zhao Q Bao DF (2021) Mycosphere notes 325–344 – Novel species and records of fungal taxa from around the world. Mycosphere 12(1): 1101–1156. 10.5943/mycosphere/12/1/14

[B27] Index Fungorum (2023) Index Fungorum. http://www.indexfungorum.org/Names/Names.asp [Accessed on 05^th^ May 2023]

[B28] Jaklitsch WM Voglmayr H (2017) Three former taxa of *Cucurbitaria* and considerations on *Petrakia* in the Melanommataceae. Sydowia 69: 81–95. 10.12905/0380.sydowia69-2017-0081 PMC5669484 29104325

[B29] Jayasiri SC Hyde KD Ariyawansa HA Bhat J Buyck B Cai L Dai YC Abd-Elsalam KA Ertz D Hidayat I Jeewon R Gareth Jones EB Bahkali AH Karunarathna SC Liu JK Luangsa-ard JJ Lumbsch HT Maharachchikumbura SSN McKenzie EHC Moncalvo JM Ghobad-Nejhad M Nilsson H Pang KL Pereira OL Phillips AJL Raspé O Rollins AW Romero AI Etayo J Selçuk F Stephenson SL Suetrong S Taylor JE Tsui CKM Vizzini A Abdel-Wahab MA Wen TC Boonmee S Dai D-Q Daranagama DA Dissanayake AJ Ekanayaka AH Fryar SC Hongsanan S Jayawardena RS Li W-J Perera RH Phookamsak R de Silva NI Thambugala KM Tian Q Wijayawardene NN Zhao RL Zhao Q Kang JC Promputtha I (2015) The Faces of Fungi database: Fungal names linked with morphology, phylogeny and human impacts. Fungal Diversity 74(1): 3–18. 10.1007/s13225-015-0351-8

[B30] Jung B Kim S Lee J (2014) Microcyle conidiation in filamentous fungi. Mycobiology 42(1): 1–5. 10.5941/MYCO.2014.42.1.1 24808726 PMC4004940

[B31] Karunarathna A Withee P Pakdeeniti P Haituk S Tanakaew N Senwanna C Działak P Karunarathna SC Tibpromma S Promthep T Monkhung S Cheewangkoon R (2022) Worldwide Checklist on Grass Fungi: What Do We Know So Far in Ascomycota. Chiang Mai Journal of Science 49(3): 742–984. 10.12982/CMJS.2022.058

[B32] Katoh K Rozewicki J Yamada KD (2019) MAFFT online service: Multiple sequence alignment, interactive sequence choice and visualisation. Briefings in Bioinformatics 20(4): 1160–1166. 10.1093/bib/bbx108 28968734 PMC6781576

[B33] Katumoto K (1980) Notes on some plant-inhabiting Ascomycotina from western Japan (1). Nippon Kingakkai Kaiho 21: 7–16.

[B34] Kobayashi T (2007) Index of fungi inhabiting woody plants in Japan. Host, Distribution and Literature. Zenkoku-Noson-Kyoiku Kyokai Publishing Co., Ltd. 1227.

[B35] Kularathnage ND Wanasinghe DN Senanayake IC Yang Y Manawasinghe IS Phillips AJL Hyde KD Dong W Song J (2022) Microfungi associated with ornamental palms: *Byssosphaeriaphoenicis* sp.nov. (Melanommataceae) and *Pseudocoleophomarhapidis* sp. nov. (Dictyosporiaceae) from south China. Phytotaxa 568(2): 149–169. 10.11646/phytotaxa.568.2.2

[B36] Li JF Phookamsak R Mapook A Boonmee S Bhat JD Hyde KD Lumyong S (2016) *Seifertiashangrilaensis* sp. nov. (Melanommataceae), a new species from Southwest China. Phytotaxa 273(1): 34–42. 10.11646/phytotaxa.273.1.3

[B37] Li JF Jeewon R Luo ZL Phookamsak R Bhat DJ Mapook A Phukhamsakda C Camporesi E Lumyong S Hyde KD (2017) Morphological characterisation and DNA based taxonomy of *Fusiconidium* gen. nov. with two novel taxa within Melanommataceae (Pleosporales). Phytotaxa 308(2): 206–218. 10.11646/phytotaxa.308.2.2

[B38] Liew ECY Aptroot A Hyde KD (2000) Phylogenetic significance of the pseudoparaphyses in Loculoascomycete taxonomy. Molecular Phylogeny and Evolution 16: 392–402. 10.1006/mpev.2000.0801 10991792

[B39] Liu XJ Guo Yl (1998) Flora Fungorum Sinicorum (Vol. 9). *Pseudocercospora*. Science Press, Beijing, 474 pp.

[B40] Lu B Hyde KD Ho WH Tsui KM Taylor JE Wong KM Yanna Zhou D (2000) Checklist of Hong Kong Fungi. Fungal Diversity Research Series, Hong Kong.

[B41] Lugato E Lavallee JM Haddix ML Panagos P Cotrufo MF (2021) Different climate sensitivity of particulate and mineral-associated soil organic matter. Nature Geoscience 14(5): 295–300. 10.1038/s41561-021-00744-x

[B42] Maheshwari R (1991) Microcycle conidiation and its genetic basis in *Neurosporacrassa*. Microbiology 137(9): 2103–2115. 10.1099/00221287-137-9-2103 1836224

[B43] Matsushima T (1980) Matsushima mycological memoirs no. 1. Saprophytic microfungi from Taiwan, part 1: Hyphomycetes 82.

[B44] Miller MA Pfeiffer W Schwartz T (2010) Creating the CIPRES Science Gateway for inference of large phylogenetic trees. Gateway Computing Environments Workshop (GCE): 1–8. 10.1109/GCE.2010.5676129

[B45] Nylander JAA Wilgenbusch JC Warren DL Swofford DL (2008) AWTY: A system for graphical exploration of MCMC convergence in Bayesian phylogenetics. Bioinformatics 24(4): 581–583. 10.1093/bioinformatics/btm388 17766271

[B46] Pan YH Guo BL Peng Y (1993) Current situation and utilization prospect of medicinal plant resources of *Hypericum* in China. Chinese Materia Medica 16: 14–18.

[B47] Pei Y (2022) Analysis of temperature variation characteristics in Zhaotong City in recent 50 years. Journal of Agricultural Catastrophology 12: 3.

[B48] Pem D Jeewon R Gafforov Y Hongsanan S Phukhamsakda C Promputtha I Doilom M Hyde KD (2019) *Melanocamarosporioidesugamica* gen. et sp. nov., a novel member of the family Melanommataceae from Uzbekistan. Mycological Progress 18(3): 471–481. 10.1007/s11557-018-1448-8

[B49] Pem D Jeewon R Chethana KWT Hongsanan S Doilom M Suwannarach N Hyde KD (2021) Species concepts of Dothideomycetes: Classification, phylogenetic inconsistencies and taxonomic standardization. Fungal Diversity 109(1): 283–319. 10.1007/s13225-021-00485-7

[B50] Priest MJ (2006) Fungi of Australia. *Septoria*. ABRS, Canberra; CSIRO Publishing, Melbourne, 259 pp.

[B51] Rambaut A (2012) FigTree v1. 4.0. A Graphical Viewer of Phylogenetic Trees. http://tree.bio.ed.ac.uk/software/figtree/ [Accessed on 16 February 2023]

[B52] Rogerson CT (1988) Pleomorphic fungi: The diversity and its taxonomic implications. Edited by Junta Sugiyama. Brittonia 40: 440. 10.2307/2807655

[B53] Roginsky VA Barsukova TK Remorova AA Bors W (1996) Moderate antioxidative efficiencies of flavonoids during peroxidation of methyl linoleate in homogeneous and micellar solutions. Journal of the American Oil Chemists’ Society 73(6): 777–786. 10.1007/BF02517955

[B54] Ronquist F Teslenko M Van Der Mark P Ayres DL Darling A Höhna S Larget B Liu L Suchard MA Huelsenbeck JP (2012) MrBayes 3.2: Efficient Bayesian phylogenetic inference and model choice across a large model space. Systematic Biology 61(3): 539–542. 10.1093/sysbio/sys029 22357727 PMC3329765

[B55] Rossman AY Crous PW Hyde KD Hawksworth DL Aptroot A Bezerra JL Bhat JD Boehm E Braun U Boonmee S Camporesi E Chomnunti P Dai DQ D’souza MJ Dissanayake A Gareth Jones EB Groenewald JZ Hernández-Restrepo M Hongsanan S Jaklitsch WM Jayawardena R Jing LW Kirk PM Lawrey JD Mapook A McKenzie EH Monkai J Phillips AJ Phookamsak R Raja HA Seifert KA Senanayake I Slippers B Suetrong S Taylor JE Thambugala KM Tian Q Tibpromma S Wanasinghe DN Wijayawardene NN Wikee S Woudenberg JH Wu HX Yan J Yang T Zhang Y (2015) Recommended names for pleomorphic genera in Dothideomycetes. IMA Fungus 6(2): 507–523. 10.5598/imafungus.2015.06.02.14 PMC4681266 26734553

[B56] Rossman AY Allen WC Braun U Castlebury LA Chaverri P Crous PW Hawksworth DL Hyde KD Johnston P Lombard L Romberg M Samson RA Seifert KA Stone JK Udayanga D White JF (2016) Overlooked competing asexual and sexually typified generic names of Ascomycota with recommendations for their use or protection. IMA Fungus 7(2): 289–308. 10.5598/imafungus.2016.07.02.09 27990336 PMC5159600

[B57] Saccardo PA (1908) Notae mycologicae. Ann Mycol 6: 553–569.

[B58] Saccardo PA (1921) Notae mycologicae. Series XXIV. I. Fungi Singaporenses Barkesiani. Bulletino dell’Orto Botanico della Regia Università di Napoli 6: 39–73.

[B59] Senanayake IC Rathnayaka AR Marasinghe DS Calabon MS Gentekaki E Lee HB Hurdeal VG Pem D Dissanayake LS Wijesinghe SN Bundhun D Nguyen TT Goonasekara ID Abeywickrama PD Bhunjun CS Jayawardena RS Wanasinghe DN Jeewon R Bhat DJ Xiang MM (2020) Morphological approaches in studying fungi: Collection, examination, isolation, sporulation and preservation. Mycosphere 11(1): 2678–2754. 10.5943/mycosphere/11/1/20

[B60] Simmonds JH (1966) Host index of plant diseases in Queensland. Host index of plant diseases in Queensland, 111 pp.

[B61] Sivanesan A Hsieh WH (1989) New species and new records of ascomycetes from Taiwan. Mycological Research 93(3): 340–351. 10.1016/S0953-7562(89)80161-3

[B62] Species Fungorum (2023) Species Fungorum. http://www.speciesfungorum.org/Names/Names.asp [Accessed on: 26^th^ June 2023]

[B63] Stamatakis A (2014) RAxML version 8: A tool for phylogenetic analysis and post-analysis of large phylogenies. Bioinformatics 30(9): 1312–1313. 10.1093/bioinformatics/btu033 24451623 PMC3998144

[B64] Swofford DL (2002) PAUP*: phylogenetic analysis using parsimony (* and other methods), version 4.0 b 10 Sinauer Associates, Sunderland, MA.

[B65] Tai FL (1979) Sylloge Fungorum Sinicorum. Sylloge fungorum Sinicorum, 1527.

[B66] Tanaka K Ooki Y Hatakeyama S Harada Y Barr ME (2005) Pleosporales in Japan (5): *Pleomassaria*, *Asteromassaria*, and *Splanchnonema*. Mycoscience 46(4): 248–260. 10.1007/S10267-005-0245-9

[B67] Teng SC (1936) V Fungi of NanKing. Contributions from the Biological Laboratory of the Science Society of China. Botanical Series 8(3): 253–270.

[B68] Tennakoon DS Kuo CH Maharachchikumbura SSN Thambugala KM Gentekaki E Phillips AJ Bhat DJ Wanasinghe DN de Silva NI Promputtha I Hyde KD (2021) Taxonomic and phylogenetic contributions to *Celtisformosana*, *Ficusampelas*, *F.septica*, *Macarangatanarius* and *Morusaustralis* leaf litter inhabiting microfungi. Fungal Diversity 108(1): 1–215. 10.1007/s13225-021-00474-w

[B69] Thiyagaraja V Hyde KD Wanasinghe DN Worthy FR Karunarathna SC (2020) Addition to Melanommataceae: A new geographical record of *Alpinariarhododendri* from Shangri La, China. Asian Journal of Mycology 3(1): 335–344. 10.5943/ajom/3/1/8

[B70] Thompson JD Gibson TJ Plewniak F Jeanmougin F Higgins DG (1997) The CLUSTAL_X windows interface: Flexible strategies for multiple sequence alignment aided by quality analysis tools. Nucleic Acids Research 25(24): 4876–4882. 10.1093/nar/25.24.4876 9396791 PMC147148

[B71] Tian Q Liu JK Hyde KD Wanasinghe DN Boonmee S Jayasiri SC Luo ZL Taylor JE Phillips AJL Bhat DJ Li WJ Ariyawansa H Thambugala KM Gareth Jones EB Chomnunti P Bahkali AH Xu JC Camporesi E (2015) Phylogenetic relationships and morphological reappraisal of Melanommataceae (Pleosporales). Fungal Diversity 74(1): 267–324. 10.1007/s13225-015-0350-9

[B72] Vu D Groenewald M De Vries M Gehrmann T Stielow B Eberhardt U Al-Hatmi A Groenewald JZ Cardinali G Houbraken J Boekhout T Crous PW Robert V Verkley GJM (2019) Large-scale generation and analysis of filamentous fungal DNA barcodes boosts coverage for kingdom fungi and reveals thresholds for fungal species and higher taxon delimitation. Studies in Mycology 92(1): 135–154. 10.1016/j.simyco.2018.05.001 29955203 PMC6020082

[B73] Wanasinghe DN Mortimer PE (2022) Taxonomic and phylogenetic insights into novel Ascomycota from forest woody litter. Biology 11(6): 889. 10.3390/biology11060889 35741409 PMC9220210

[B74] Wanasinghe DN Jones EBG Dissanayake AJ Hyde KD (2016) Saprobic Dothideomycetes in Thailand: *Vaginatisporaappendiculata* sp. nov. (Lophiostomataceae) introduced based on morphological and molecular data. Studies in Fungi 1(1): 56–68. 10.5943/sif/1/1/5

[B75] Wanasinghe DN Phukhamsakda C Hyde KD Jeewon R Lee HB Jones EBG Tibpromma S Tennakoon DS Dissanayake AJ Jayasiri SC (2018) Fungal diversity notes 709–839: Taxonomic and phylogenetic contributions to fungal taxa with an emphasis on fungi on Rosaceae. Fungal Diversity 89(1): 1–236. 10.1007/s13225-018-0395-7

[B76] Wanasinghe DN Wijayawardene NN Xu J Cheewangkoon R Mortimer PE (2020) Taxonomic novelties in *Magnolia*-associated pleosporalean fungi in the Kunming Botanical Gardens (Yunnan, China). PLoS ONE 15(7): e0235855. 10.1371/journal.pone.0235855 PMC7357747 32658904

[B77] Wanasinghe DN Mortimer PE Bezerra JDP (2022) Editorial: Fungal Systematics and Biogeography. Frontiers in Microbiology 12: 827725. 10.3389/fmicb.2021.827725 PMC8822041 35145501

[B78] Wei CT Huang SW (1939) A checklist of fungi deposited in the Mycological Herbarium of the University of Nanking. Nanking Journal 9: 329–372.

[B79] White RP Murray S Rohweder M Prince SD Thompson KM (2000) Grassland ecosystems. World Resources Institute, Washington, D.C., USA, 81.

[B80] Wijayawardene NN Hyde KD Wanasinghe DN Papizadeh M Goonasekara ID Camporesi E Bhat DJ McKenzie EHC Phillips AJL Diederich P Tanaka K Li WJ Tangthirasunun N Phookamsak R Dai DQ Dissanayake AJ Weerakoon G Maharachchikumbura SSN Hashimoto A Matsumura M Bahkali AH Wang Y (2016) Taxonomy and phylogeny of dematiaceous coelomycetes. Fungal Diversity 77(1): 1–316. 10.1007/s13225-016-0360-2

[B81] Wijayawardene NN Hyde KD Rajeshkumar KC Hawksworth DL Madrid H Kirk PM Braun U Singh RV Crous PW Kukwa M Lücking R Kurtzman CP Yurkov A Haelewaters D Aptroot A Lumbsch HT Timdal E Ertz D Etayo J Phillips AJL Groenewald JZ Papizadeh M Selbmann L Dayarathne MC Weerakoon G Jones EBG Suetrong S Tian Q Castañeda Ruíz RF Bahkali AH Pang KL Tanaka K Dai DQ Sakayaroj J Hujslová M Lombard L Shenoy BD Suija A Maharachchikumbura SSN Thambugala KM Wanasinghe DN Sharma BO Gaikwad S Pandit G Zucconi L Onofri S Egidi E Raja HA Kodsueb R Cáceres MES Pérez-Ortega S Fiuza PO Monteiro JS Vasilyeva LN Shivas RG Prieto M Wedin M Olariaga I Lateef AA Agrawal Y Fazeli SAS Amoozegar MA Zhao GZ Pfliegler WP Sharma G Oset M Abdel-Wahab MA Takamatsu S Bensch K de Silva NI De Kesel A Karunarathna A Boonmee S Pfister DH Lu YZ Luo ZL Boonyuen N Daranagama DA Senanayake IC Jayasiri SC Samarakoon MC Zeng XY Doilom M Quijada L Rampadarath S Heredia G Dissanayake AJ Jayawardana RS Perera RH Tang LZ Phukhamsakda C Hernández-Restrepo M Ma X Tibpromma S Gusmao LFP Weerahewa D Karunarathna SC (2017) Notes for genera – Ascomycota. Fungal Diversity 86(1): 1–594. 10.1007/s13225-017-0386-0

[B82] Wijayawardene NN Hyde KD Dai DQ Sánchez-García M Goto BT Saxena RK Erdoğdu M Selçuk F Rajeshkumar KC Aptroot A Błaszkowski J Boonyuen N da Silva GA de Souza FA Dong W Ertz D Haelewaters D Jones EBG Karunarathna SC Kirk PM Kukwa M Kumla J Leontyev DV Lumbsch HT Maharachchikumbura SSN Marguno F Martínez-Rodríguez P Mešić A Monteiro JS Oehl F Pawłowska J Pem D Pfliegler WP Phillips AJL Pošta A He MQ Li JX Raza M Sruthi OP Suetrong S Suwannarach N Tedersoo L Thiyagaraja V Tibpromma S Tkalčec Z Tokarev YS Wanasinghe DN Wijesundara DSA Wimalaseana SDMK Madrid H Zhang GQ Gao Y Sánchez-Castro I Tang LZ Stadler M Yurkov A Thines M (2022a) Outline of Fungi and fungus-like taxa – 2021. Mycosphere 13(1): 53–453. 10.5943/mycosphere/13/1/2

[B83] Wijayawardene NN Phillips AJL Pereira DS Dai DQ Aptroot A Monteiro JS Druzhinina IS Cai F Fan X Selbmann L Coleine C Castañeda-Ruiz RF Kukwa M Flakus A Fiuza PO Kirk PM Kumar KCR leperuma Arachchi IS Suwannarach N Tang L-Z Boekhout T Tan CS Jayasinghe RPPK Thines M (2022b) Forecasting the number of species of asexually reproducing fungi (Ascomycota and Basidiomycota). Fungal Diversity 114(1): 463–490. 10.1007/s13225-022-00500-5

[B84] Wu J Yu N Peng D Xing S (2021) The partial chloroplast genome of *Hypericummonogynum* L. (Guttiferae). American Journal of Plant Sciences 12(5): 707–710. 10.4236/ajps.2021.125047

[B85] Xi QN Lin KH Wei JH (2007) Advances on chemical investigation of *Hypericum*. Journal of Natural Product Research and Development 19: 344–355. [In Chinese]

[B86] Yuan ZQ Barr ME (1994) New ascomycetous fungi on bush cinquefoil from Xinjiang, China. Sydowia 46(2): 329–337.

[B87] Yuan W Jiuming H Hui C Disheng Z Hua C Huibo S (2006) Analysis of flavones in *Rubusparvifolius* Linn by high performance liquid chromatography combined with electrospray ionisation-mass spectrometry and thin-layer chromatography combined with Fourier transform surface enhanced Raman spectroscopy. Chinese Journal of Analytical Chemistry 34(8): 1073–1077. 10.1016/S1872-2040(06)60050-9

[B88] Zeng YR Wang LP Hu ZX Yi P Yang WX Gu W Huang LJ Yuan CM Hao XJ (2018) Chromanopyrones and a flavone from *Hypericummonogynum*. Fitoterapia 125: 59–64. 10.1016/j.fitote.2017.12.013 29269232

[B89] Zhang Z (2006) Flora Fungorum Sinicorum. *Botrytis*, *Ramularia*. Science Press, Beijing, 26: 277.

[B90] Zhang S Peng G Xia Y (2010) Microcycle conidiation and the conidial properties in the entomopathogenic fungus *Metarhiziumacridum* on agar medium. Biocontrol Science and Technology 20(8): 809–819. 10.1080/09583157.2010.482201

[B91] Zhu H Shen YX Chen FJ Fu X Wang W (2022) Impacts of the degraded grassy hill and artificial reconstruction on soil seed bank in southern Zhaotong. Acta Agrestia Sinica 30: 1359–1369. 10.11733/j.issn.1007-0435.2022.06.006

[B92] Zhuang WY (2001) Higher Fungi of Tropical China. *Mycotaxon*, Ltd., Ithaca, NY, 485 pp.

[B93] Zhuang WY (2005) Fungi of northwestern China. *Mycotaxon*, Ltd., Ithaca, NY, 430 pp.

[B94] Zhuang JY (2012) Flora Fungorum Sinicorum: Uredinales (IV). Science Press, Beijing, 41: 254.

